# The *Xenopus* ORFeome: A resource that enables functional genomics

**DOI:** 10.1016/j.ydbio.2015.09.004

**Published:** 2015-12-15

**Authors:** Ian M. Grant, Dawit Balcha, Tong Hao, Yun Shen, Prasad Trivedi, Ilya Patrushev, Joshua D. Fortriede, John B. Karpinka, Limin Liu, Aaron M. Zorn, P. Todd Stukenberg, David E. Hill, Michael J. Gilchrist

**Affiliations:** a The Francis Crick Institute, Mill Hill Laboratory, The Ridgeway, Mill Hill, London NW7 1AA, UK; b Center for Cancer Systems Biology (CCSB) and Department of Cancer Biology, Dana-Farber Cancer Institute, Boston, MA 02215, USA; c Xenbase, Division of Developmental Biology, Cincinnati Children's Hospital Medical Center, Cincinnati, OH 45229, USA; d Xenbase, Department of Biological Science, University of Calgary, Calgary, AB, Canada; e University of Virginia, School of Medicine, Charlottesville, VA 22908, USA

**Keywords:** Gateway, Recombinational cloning, ORFeome, *Xenopus*, Open reading frame, Gene annotation

## Abstract

Functional characterisation of proteins and large-scale, systems-level studies are enabled by extensive sets of cloned open reading frames (ORFs) in an easily-accessible format that enables many different applications. Here we report the release of the first stage of the *Xenopus* ORFeome, which contains 8673 ORFs from the *Xenopus* Gene Collection (XGC) for *Xenopus laevis*, cloned into a Gateway® donor vector enabling rapid in-frame transfer of the ORFs to expression vectors. This resource represents an estimated 7871 unique genes, approximately 40% of the non-redundant *X. laevis* gene complement, and includes 2724 genes where the human ortholog has an association with disease. Transfer into the Gateway system was validated by 5′ and 3′ end sequencing of the entire collection and protein expression of a set of test clones. In a parallel process, the underlying ORF predictions from the original XGC collection were re-analysed to verify quality and full-length status, identifying those proteins likely to exhibit truncations when translated. These data are integrated into Xenbase, the *Xenopus* community database, which associates genomic, expression, function and human disease model metadata to each ORF, enabling end-users to search for ORFeome clones with links to commercial distributors of the collection. When coupled with the experimental advantages of *Xenopus* eggs and embryos, the ORFeome collection represents a valuable resource for functional genomics and disease modelling.

## Highlights

• The Xenopus ORFeome, v1.0, containing 8673 ORFs from the *Xenopus* Gene Collection.
• 7871 genes, ∼40% of the *X. laevis* proteome, 2724 genes with human disease orthologs.
• XGC ORFs re-analysed via improved bioinformatics approaches, 85% verified full-length.
• Information on ORFeome clones can be found on Xenbase.org, with links to distributors.
• A valuable resource for accelerating functional genomics and human disease modelling.

## 1 Introduction

*Xenopus* is a powerful vertebrate model system for investigating protein function and it has a rich history of functional genomics (Harland and Grainger, 2011). There are currently two members of the genus used for biomedical research: the earlier adopted, allotetraploid *Xenopus laevis*, and its slightly smaller cousin *Xenopus tropicalis*, which has a normal diploid DNA complement. Both species are widely used, and share the key characteristic of large, abundant, externally developing eggs and embryos, making them ideally suited for the discovery and analysis of protein function. For example, many well-known developmental regulators such as Noggin (Smith and Harland, 1992) and Dickkopf (Glinka et al., 1998) were first identified by functional screening of synthetic mRNA from cDNA libraries injected into *Xenopus* embryos. Moreover, essential cell cycle regulators such as INCENP (Stukenberg et al., 1997), Securin (Zou et al., 1999), Geminin (McGarry and Kirschner, 1998) and Sororin (Rankin et al., 2005) were identified and characterized using functional screens in *Xenopus* extracts. A key reagent for these types of screens and the analysis of protein function in *Xenopus* are high quality cDNA libraries.

Over the last decade there were two major efforts to generate large-scale cDNA collections with high diversity and low redundancy as resources for the community: the NIH-sponsored IMAGE Consortium *Xenopus* Gene Collection (XGC), in collaboration with the Joint Genome Institute and many individual labs (Klein et al., 2002); and the Wellcome Trust-funded *X. tropicalis* EST sequencing project run jointly by the Sanger and Gurdon Institutes in the UK (Gilchrist et al., 2004). These projects took similar approaches by performing large scale sequencing on diverse cDNA libraries followed by computational identification of full-length clones and re-arraying to create non-redundant full-length collections (reviewed in Gilchrist (2012)). Clones from both projects were made available through commercial distributors, and have clearly influenced ongoing research efforts using this model system: they are currently cited in PubMed Central in 52 and 49 articles respectively. Although these resources continue to be useful, the rate-limiting step in contemporary research can often be subcloning of the open reading frames (ORFs) into different types of expression plasmids (e.g. CMV or SP6 promoters) with the appropriate epitope tags (e.g. GFP, flag, etc.) for the many different types of functional experiments that researchers perform. To address this need, and facilitate functional genomics in *Xenopus*, we therefore set out to generate an ORFeome (Brasch et al., 2004) for the *Xenopus* model system, comprising a large collection of *Xenopus* ORFs in a Gateway-compatible vector (Hartley et al., 2000, Hartley et al., 2000; Walhout et al., 2000) that enables rapid and easy transfer to many different plasmids using the bacterial recombination system.

A Gateway-based ORFeome is designed to streamline the generation of cDNA clones amenable to use for high-throughput expression of protein, including the removal of untranslated regions, mutation of the stop codon (in order to generate 3′ fusion proteins), and engineering 5′ and 3′ ends so that they have suitable restriction or recombineering sequences. Moving the cDNA into a general purpose Gateway entry vector has the added utility that the clone can subsequently be easily moved into a variety of destination vectors with distinct uses. The Gateway system utilises the bidirectional, site-specific recombination of bacteriophage *λ*; the in vitro reactions are efficient, offer nucleotide precision, and enable large-scale automation of unidirectional cloning of ORFs into a wide array of plasmids for functional experiments. ORFs in Gateway entry constructs can be transferred between vectors in a matter of hours, rather than the 2–3 days typically required by conventional cloning methods. Different types of destination vector allow expression of ORFs in bacteria, eukaryotic cells, or, in the case of the pCS2 family of vectors, enable the production of synthetic mRNA for injection into *Xenopus* oocytes and embryos (Rupp et al., 1994). Destination vectors are also frequently used to enable the addition of N- or C-terminal fusions such as green fluorescent protein (GFP) or small epitope tags (e.g. HA or Flag).

ORFeomes using the Gateway recombination cloning system have previously been constructed for a range of species, including: human (Lamesch et al., 2007, Rual et al., 2004, Yang et al., 2011), *Caenorhabditis elegans* (Reboul et al., 2003), *Saccharomyces cerevisiae* (Gelperin et al., 2005), *Brucella melitensis* (Dricot et al., 2004), *Chlamydophila pneumoniae* (Maier et al., 2012), *Staphylococcus aureus* (Brandner et al., 2008), *Escherichia coli* (Rajagopala et al., 2010), as well as being partially available for *Drosophila melanogaster* (Bischof et al., 2013), and mouse (Temple et al., 2009). See Table 1 for a summary of methods and coverage.

The development of a *Xenopus* ORFeome is an important part of keeping the research capabilities of this model system up to date, and will enable researchers to create the right tools to do this. Although protein function is often conserved, scientific rigour requires that *Xenopus* experiments are performed with *Xenopus* proteins. Proteins co-evolve, and the necessary tight interactions between species-specific proteins may be less effective between proteins that have evolved separately. In addition this is an opportunity to generate an ORFeome where every ORF has been analysed for completeness and the analysis is transparently presented. Furthermore, the data generated in the production of the ORFeome will improve our definition of the transcriptome and hence the annotation of gene loci on the genome; these will be integrated into Xenbase, the *Xenopus* community database (Bowes et al., 2008, Karpinka et al., 2014).

This project has two phases: the first phase will take advantage of the existing full-length cDNA resources for each *Xenopus* species, and the second phase will apply a more experimentally intensive, RT-PCR based method using predicted ORFs. Phase one will proceed in two stages, starting with the *X. laevis* clones from the XGC collection (which we are currently releasing, and describe here), before moving on to the *X. tropicalis* clones from both the XGC and Wellcome/Sanger collections. This order is determined in part by practical considerations, as the XGC *X. laevis* collection covers more genes than either of the *X. tropicalis* collections (Gilchrist, 2012), and because *X. laevis* is currently the more widely used of the two frog model species, and was therefore set as the first priority by the community.

Here we describe the generation and validation of the *Xenopus* ORFeome, version 1.0, which contains 8673 *X. laevis* ORFs from the NIH *Xenopus* Gene Collection (XGC) (http://xgc.nci.nih.gov/) (Klein et al., 2002), and includes 2724 genes where the human ortholog has an association with disease. The *Xenopus* ORFeome collection will transform the ability of the community both to rapidly characterize protein function, and to screen for novel activities in this highly effective disease model. The ORFeome collection will be made available to the community through a number of distributors, and may be purchased as individual clones or complete sets. Information on the *Xenopus* ORFeome collection can be found at http://www.xenbase.org/reagents/static/orfeome.jsp.

## 2 Materials and methods

### 2.1 Gateway cloning of *X. laevis* open reading frames from the *Xenopus* Gene Collection

The *Xenopus* Gene Collection (XGC) resource (Klein et al., 2002) is made available through the IMAGE Consortium network (http://xgc.nci.nih.gov/), and for *X. laevis* comprises 104 96-well plates containing 9984 putatively full-length cDNA clones. From a copy of this we extracted 9805 viable cDNA clones, which therefore comprised our starting material. The original clones were collected containing both coding sequence and 5′ and 3′ untranslated regions (UTRs), and the XGC reference open reading frames varied in length from 75 bp to 7.5 kb. We attempted to clone all the available ORFs for which we had viable starting material.

**Fig. 1:**
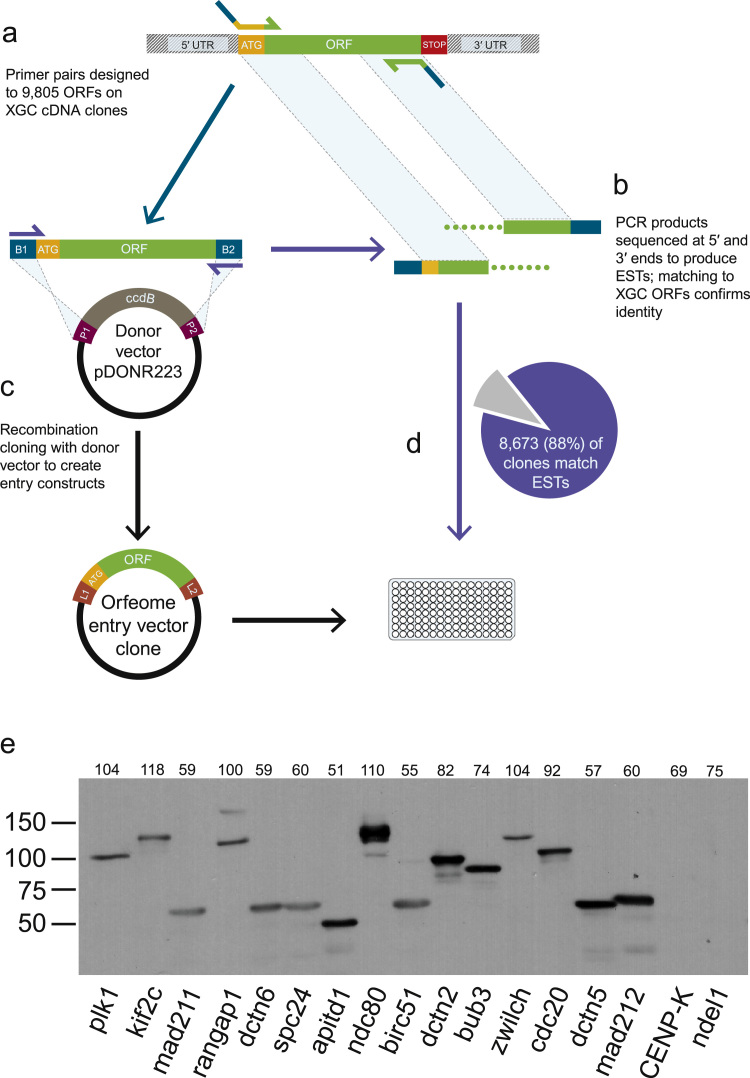
Schematic overview of generation of the *Xenopus* ORFeome, v1.0. (a) PCR primers were designed to 9805 ORFs from the XGC cDNA library. (b) The PCR products were EST sequenced at 5′ and 3′ ends, and these sequences were BLASTed against the XGC ORFs. (c) BP recombination cloning reaction with the donor vector, pDONR223 gives the bare ORF in a Gateway entry construct. (d) 8673 Gateway entry clones were consolidated based on confirmation of identity from sequencing to produce *Xenopus* ORFeome v1.0. (e) Imunoblots of 17 proteins after transfer from *Xenopus* ORFeome Gateway entry vectors into a C-terminal GFP-tagged destination vector after in vitro translation. The different proteins act as internal controls for each other in this experiment, success is indicated by the relative positions of bands of predicted size, compared to standards (Bio-Rad). Weights are given in kDa, and include 36 kDa for the LAP tag (GFP-TEV-S) fusion. Faint bands may be caused by ribosomes starting at incorrect start sites, or from post-translational modifications.

The strategy for cloning the ORFs from the XGC cDNA library into Gateway entry vectors for the *Xenopus* ORFeome (see Fig. 1) is identical to that developed for use in the human ORFeome, version 1.1 (Rual et al., 2004). Briefly, this involves polymerase chain reaction (PCR) amplification of the open reading frame portion of an XGC cDNA clone using a pair of ORF-specific primers for each clone. Primers were designed from the set of XGC reference ORFs as supplied. Forward primers were designed from the first base of the start codon, and reverse primers were designed from the last base preceding the stop codon, omitting the stop codon from subsequent Gateway constructs. Forward and reverse ORF-specific primers were extended with Gateway attB1 and attB2 sites, respectively, to facilitate easy recombination cloning via a Gateway BP reaction with pDONR223 as described (Rual et al., 2004). Plasmids were introduced into chemically competent DH5α bacteria, selecting for spectinomycin resistance.

### 2.2 Clone sequencing and consolidation

PCR products from the Gateway clones were Sanger-sequenced from both ends to generate 5′ and 3′ expressed sequence tags (ESTs) (Reboul et al., 2001). An existing software pipeline (Lamesch et al., 2007, Reboul et al., 2001, Rual et al., 2004) was used to verify that the cloned ORF contained the expected sequence: ESTs were aligned against a database of the reference ORF coding sequences from the XGC set, and the best hit recorded for each EST. An ORF was considered to have been successfully cloned if at least one of the ESTs matched the reference sequence with a BLAST score (Altschul et al., 1990) of 50 or more. Successful clones were consolidated into a new array of 93 96-well plates to form the *Xenopus* ORFeome, version 1.0.

### 2.3 Validation of Gateway transferability of *Xenopus* ORFeome clones

Twenty ORFs of various molecular weights and functions, that passed the Gateway cloning QC criteria for inclusion in the *Xenopus* ORFeome v1.0 collection, were transferred from their Gateway entry vectors via an LR reaction into the pCSf107mT-Gateway-3′LAP destination vector (see following), which contains a green fluorescent protein (GFP) tag at the C-terminal end of the ORF. Following LR transfer, clone identity and in-frame transfer were confirmed by Sanger sequencing. Three clones failed at this stage. Sequence-confirmed clones were then expressed in a coupled, in vitro transcription and translation reaction in reticulocyte lysate, and the product identified by immunoblot using anti-GFP antibodies.

### 2.4 Generation of the pCSf107mT-Gateway-3′LAP tag vector

A LAP tag contains enhanced green fluorescent protein (eGFP) followed by a tobacco etch virus (TEV) protease site and an S-tag for affinity purification (Raines et al., 2000). The LAP tag from the pIC113 vector (Cheeseman and Desai, 2005) was sub-cloned into the pcDNA5.0/FRT vector (Invitrogen) using the Xho I site to generate the pcDNA5.0/FRT-LAP-N vector. The CmR-ccdB cassette from the pDONR221 vector was amplified via PCR and inserted upstream of the LAP tag of the pcDNA5.0/FRT-LAP-N vector using the Kpn I site to make the DLAP-C vector. To make the pCSf107mT-Gateway-3′LAP destination vector, the CmR-ccdB cassette and LAP tag from the DLAP-C vector was amplified via PCR and inserted into the pCSf107mT vector (Mii and Taira, 2009) using the Bgl II and Xho I sites. Plasmids containing this destination vector (and three others: pCSf107mT-Gateway-3′ Flag, pCSf107mT-Gateway-3′ 3xHA and pCSf107mT-Gateway-3′ Myc) may be obtained from Addgene (www.addgene.org) under the IDs 67616, 67617, 67618 and 67619.

### 2.5 Tests for computational verification of the XGC reference ORFs

Using the NCBI BLAST+ alignment tool (Camacho et al., 2009), the full cDNA sequence of each XGC cDNA clone, including the 5′ and 3′ UTRs, was aligned to cluster consensus sequences from the *X. laevis* full-length EST database (Gilchrist et al., 2004) (http://genomics.nimr.mrc.ac.uk/online/xt-fl-db.html, assembly Xl4). To find the correct homeolog we noted the best-matching EST cluster for each XGC cDNA, using a percent identity match cut-off of 95% and ordering the alignments by start coordinate on the reference sequence and then BLAST bit-score. Where there were no suitable matches, we ran a second search using a lower percent identity cut-off (80%) to find a match with the alternate homeolog.

**Fig. 2:**
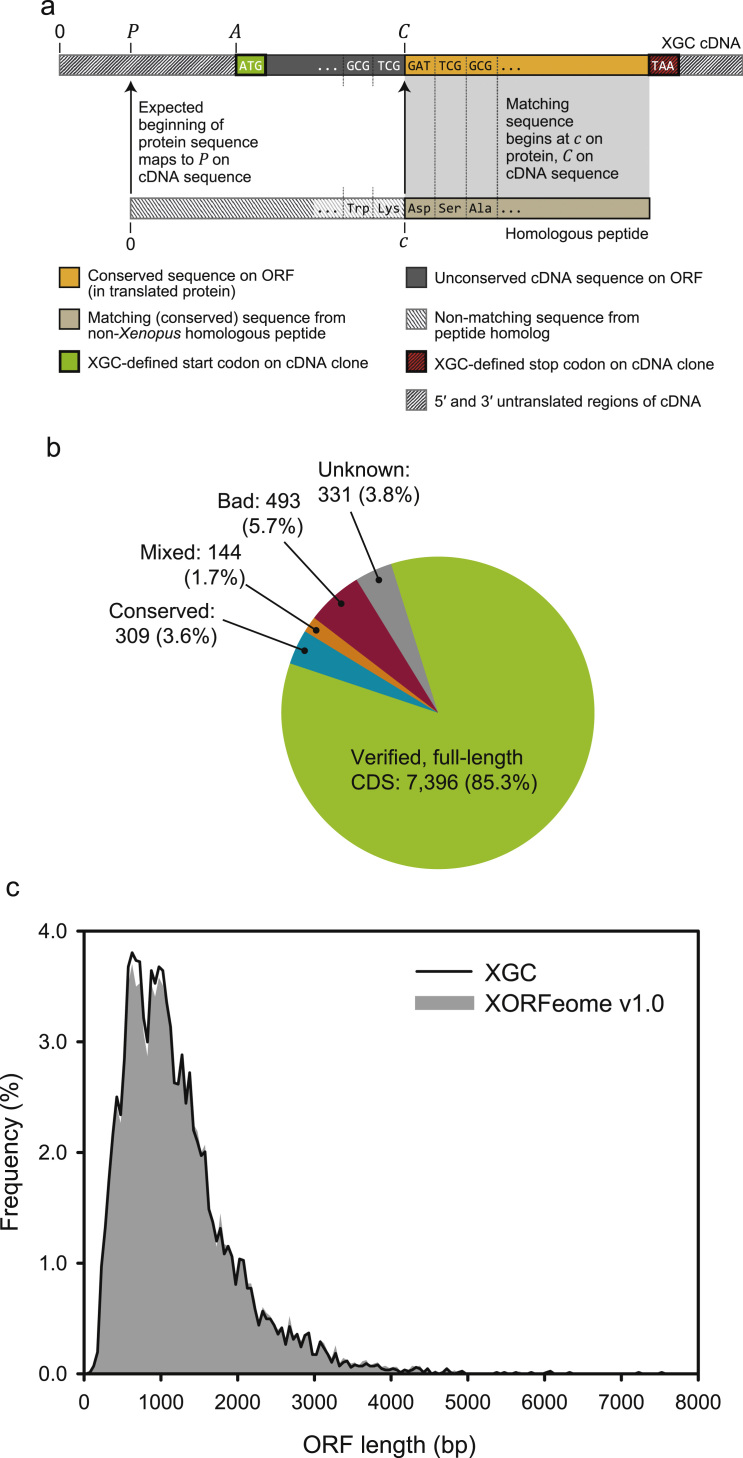
Testing the full-length status of the XGC ORFs (a) using protein alignment: translated protein alignments to non-*Xenopus* species yields a predicted position for the start codon on a zero-based scale running from 5′ to 3′ along the length of the cDNA sequence. The position of the XGC-defined start codon on the clone cDNA sequence is marked as A. The start of conserved protein alignment on the XGC cDNA sequence is labelledC, and P is the predicted position of the start codon based on the length of the protein sequence, i.e. P=C−c, where c is the position of the start of conservation on the non-*Xenopus* protein sequence. The minimum possible value of C is zero, but P can be negative. For each XGC ORF, P and C were averaged over all the respective homologous protein alignments to produce single, average values for $\bar{P}$ and $\bar{C}$, respectively. (b) Analysis of the XGC ORFs, over all tests, predicts 85% of the *Xenopus* ORFeome v1.0 to be full-length (89% including *conserved* ORFs, see Section 2). (c) Frequency distribution of open reading frame lengths in the source XGC cDNA library and the consolidated *Xenopus* ORFeome collection, showing equal likelihood of cloning long and short ORFs.

The NCBI BLASTX tool was used to find the best matching protein for each XGC cDNA clone from each of six selected non-*Xenopus* species: human (*Homo sapiens*), mouse (*Mus musculus*), chicken (*Gallus gallus*), zebrafish (*Danio rerio*), worm (*S. cerevisiae*), and fly (*D. melanogaster*). Averaging over these alignments, the relative position on the XGC cDNA sequence of the predicted N-terminus of a conserved protein was calculated (Fig. 2a). These data were then combined to score each reference ORF according to a series of tests (Table 2).

An additional pair of tests was created by taking short (96 bp) sequence tags from both the start of the XGC reference ORF, and from successive in-frame ATG positions within the open reading frames defined in the assembled EST contigs described above. These latter are labelled ATG1, ATG2, etc., and are then re-labelled using the full-length scores with positional information associated with these assemblies, in particular the BLH/ATG score (Gilchrist et al., 2004) indicating the most likely ATG to be a conserved start of translation from a basket of species' proteins, and the EST/CLI, or *cliff*, score (Gilchrist et al., 2015) indicating the likely start of *transcription*. If the position of the BLH/ATG score is at the position of a tag labelled ‘ATG1’, the tag is re-labelled ‘ORF’, or if it is at the position of a higher number ‘ATG*n*’ (n≤9) it is re-labelled ‘CONS’ (conserved); scores below 10 are ignored. If the position of the EST/CLI score is before, or within 12 bp, in the 3′ direction, of a tag labelled ATG1, then the tag is re-labelled ‘CLI’; ‘ORF’ is preferred over ‘CLI’. The XGC reference tags are then matched with all the EST contig tags to find the best match, with at least 80% similarity, and the label on the EST tag is used to evaluate the XGC ORF. Labels ‘ORF’, ‘CONS’, CLI’ and ‘ATG1’ indicate a full-length or conserved N-terminus protein; conversely, labels ‘ATG2’, ‘ATG3’, etc. indicated a truncated protein.

The ORFs were then grouped into five categories according to the sum of these tests (Table 3). A positive tag test ‘CONS’, indicating a conserved N-terminus within a slightly truncated open reading frame, is used to over-ride negative test indications.

### 2.6 Application of Xenbase gene annotation to the XGC ORFs

Using the NCBI BLAST+ tool, the XGC open reading frames were matched to mRNA sequences provided by the *Xenopus* community database resource, Xenbase, which have pre-existing stable Xenbase gene IDs assigned to them (Bowes et al., 2008, Karpinka et al., 2014). Mapping tables were also provided by Xenbase to associate gene names and symbols to the IDs. Another Xenbase mapping table groups individual genes together under an umbrella gene ID where they are known to be homeologs of one another in *X. laevis*, or orthologs of the related gene in *X. tropicalis*. (Genes may generally be duplicated in the allotetraploid *X. laevis* compared to the diploid *X. tropicalis*, and these duplicated genes are referred to as homeologs.)

### 2.7 Human disease gene orthology

To establish relationships between the ORFeome clones and human disease-associated genes, we took data from a number of sources. First we downloaded the list of human genes linked to disease phenotypes from the *Online Mendelian Inheritance in Man* database (OMIM, http://omim.org); these were linked through the MIM gene IDs reported on Xenbase gene pages to *Xenopus* mRNA sequences, and these were linked via BLAST similarity to the XGC reference sequence associated with each ORFeome clone. This created a set of *direct* associations between human disease genes and their *Xenopus* orthologs. In addition we downloaded data from a published, large-scale database of inferred human protein complexes (Lage et al., 2008), where each complex contained at least one gene–disease link from OMIM. Proteins in these disease-associated complexes, but without a direct link to OMIM, were considered to have an *indirect* association with the disease or diseases associated with each complex. The data downloaded from this source comprised MIM disease phenotypes, Ensembl human protein IDs and protein complex identifiers; these were linked via Ensembl/NCBI-Entrez ID conversions, downloaded from Biomart, to the Entrez ID of the human ortholog stored on Xenbase gene pages, and then linked as above from the *Xenopus* mRNAs to the ORFeome clones. The union of these two datasets was used to identify human disease orthology within the *Xenopus* ORFeome, and in addition we recorded whether the gene-disease association was direct (via OMIM) or indirect (via Lage et al. (2008)).

## 3 Results and discussion

### 3.1 Generation of the Gateway-compatible *X. laevis* ORFeome collection

In order to generate the first version of the *Xenopus* ORFeome, we took advantage of the *Xenopus* Gene Collection (XGC) resource (http://xgc.nci.nih.gov/), a trans-NIH initiative that collated a set of 9984 putatively full-length *X. laevis* cDNA clones from a number of cDNA libraries derived from different tissues and developmental stages produced as part of the *Xenopus* EST project (Klein et al., 2002). These XGC clones contain both coding sequence and 5′ and 3′ untranslated regions (UTRs).

We were aware at the start of the project, given an earlier analysis of part of the *X. tropicalis* XGC clone data (Gilchrist, 2012), that there may be errors in the *X. laevis* XGC reference ORFs. However, as the funding for the *Xenopus* ORFeome was based in part on a mandate from the community as the highest ranked *immediate need* in the 2011 *Xenopus* White paper (http://www.xenbase.org/community/static/xenopuswhitepaper/2011/XWP_xenbase.pdf), we felt it was important to make a prompt start with the cloning. We reasoned that bioinformatics analysis of the reference ORFs could be done at a later date (see below), and although this would likely mean inclusion of some truncated clones in the Gateway collection, this was preferable to a delay of twelve months or more whilst bioinformatics resources were put in place and analysis methods developed.

High fidelity PCR was used to amplify putative ORFs from the XGC clones, using the XGC reference open reading frame coordinates, beginning with the start codon but omitting the stop codon. These products were then introduced into the pDONR223 Gateway vector as described (see Section 2). The resulting *Xenopus* entry clone plasmids are propagated as bacterial transformants.

We successfully cloned open reading frames from 8673 (88%) of the 9805 clones recovered as starting material from our copy of the XGC collection (see Section 2). This compares favourably with both the human ORFeome, v8.1 (Yang et al., 2011), and the *C. elegans* ORFeome, v1.1 (Reboul et al., 2003), with recovery rates of 84% and 62%, respectively. The median open reading frame lengths in the successfully cloned ORFs compared to the reference ORFs in the whole XGC collections are 1053 bp and 1080 bp, respectively. In addition, there appears to be no significant difference between the two distributions of ORF lengths (Fig. 2c), indicating that longer open reading frames are not significantly more likely to fail during the cloning process than shorter ones, contrary to the behaviour observed in a previous high throughput Gateway cloning project (Reboul et al., 2003). Successfully cloned ORFs were consolidated into a new set of distribution plates. The plate map of the *Xenopus* ORFeome is provided as Supplementary information Table S1, and contains the plate and well location of each clone, its full-length status (see below), the gene name, and other relevant information.

### 3.2 Validation of Gateway transferability of *Xenopus* ORFeome clones

To validate the performance of the finished Gateway entry clones, we selected 20 clones from those which passed the quality control tests of the Gateway cloning process, and would therefore be part of the *Xenopus* ORFeome v1. These were then transferred using standard methods from the initial Gateway entry vectors into a pCSf107mT-Gateway-3′LAP destination vector (see Section 2). Two clones failed outright at this step, and appear not to have inserts; these may have been deleted by the host as toxic. A third clone contained the correct sequence, but in a mixture of other sequences, which we were able to resolve by streaking out single colonies and sequencing five of these: two out of five were correct. We suggest that this may be good standard practise for handling this material. The remaining 17 clones were verified by sequencing to have been correctly transferred to the destination vector, and these were then further characterized through coupled, in vitro transcription and translation (see Section 2). Fifteen of these gave immunoblot products of the expected molecular weight (Fig. 1e).

Given the sequence verification, it is unclear why the other two proteins were not correctly translated. It is possible that the protein products are degraded. We know that CenpK protein is a subunit of a large kinetochore complex (Cenp-HIKM) and depletion of any protein in the complex results in co-depletion of the other subunits from kinetochores (Cheeseman et al., 2008). We also note that Ndel1 exists in a complex, so it is possible that production of these proteins in an inappropriate molecular environment results in their rapid degradation. Nevertheless, we conclude that a random set of clones from the *Xenopus* ORFeome could be transferred to a destination vector with a reasonable success rate, and if our test set is representative this would be approximately 85%. The failure of transferred clones to generate stable protein is likely to be protein specific, and users may wish to test that generated proteins are detectable and of the correct molecular weight.

### 3.3 Gene coverage in the ORFeome

Estimating gene coverage is slightly complicated by the allotetraploid nature of *X. laevis*. Duplicated genes (termed homeologs) are considered to have been orthologs of each other in the two (presumed) ancestral diploid frogs, and are therefore both orthologous to the equivalent single gene in the diploid *X. tropicalis*. In order to simplify discussion of gene numbers here, and although we recognise that some of the homoleogous pairs may have become sub-functionalized, we refer to *diploid equivalent genes*, where two homeologs count as a single gene.

To determine how many diploid equivalent genes are covered by the 8402 distinct ORFs in the *Xenopus* ORFeome, we used annotation from Xenbase. Each of the ORFs has an assigned Xenbase gene ID, and they are divided into two groups: 6946 ORFs, within which homeologous relationships have been identified through curation, and 1456 ORFs where the homeologous relationships are not known. The curated group covers 6507 (diploid equivalent) gene loci; and from this we can estimate that the uncurated group likely covers 1364 gene loci (assuming a similar proportion of homeologous pairs). The whole ORFeome therefore covers an estimated 7871 diploid equivalent *Xenopus* genes, containing 531 homeologous pairs, with an additional 271 duplicated ORFs.

Although it is widely accepted that the inter-specific hybridisation event giving rise to whole genome duplication in *X. laevis* will likely have been followed by gene loss, it is interesting that we only identify clones for both homeologs in ∼6.7% of the genes on our collection. This is, however, in proportion to the total numbers of homeologous pairs identified on Xenbase (of 12532 gene pages with at least on *X. laevis* gene, only 836 have two identified homeologs), and it is not clear to what extent these numbers simply reflect current annotation efforts, or whether this tells us something more interesting about the *expression* of homeologous genes. At the DNA level, a recent study found separate hybridisation signals on homeologous pairs of *X. laevis* chromosomes for 50/60 (80%) selected genes (Uno et al., 2013), and direct estimates of the sizes of homeologous chromosome pairs found that one of each pair is smaller than the other by factors between 0.75 and 0.94 (Matsuda et al., 2015), suggesting an approximate upper bound for gene retention if gene loss is proportionate to sequence loss.

### 3.4 Verification of the XGC reference ORFs

Towards the end of the physical cloning process, and as the necessary resources became available, we performed computational analysis of the XGC reference ORFs on which the cloning was based. Our aim was to provide a comprehensive analysis of the ORFeome collection, giving users clear guidance as to which clones are highly likely to contain the complete coding sequence, and which may be truncated. Such analyses have not been previously reported for ORFeome collections of other species, but, anecdotally, truncated clones have been observed in collections made to date. It is challenging to accurately predict the true start codon of an open reading frame in single pass sequence data from large clone collections, and independent analysis of such data (Gilchrist, 2012) suggests that around 10% are likely to be misidentified as full-length. Information on the likelihood that a given clone is a *bona fide* full-length clone would therefore be very valuable.

To investigate this, we devised a series of tests of the XGC sequences based on three sets of data: BLASTn alignments between the XGC reference sequences and the consensus sequences from assembled EST contigs with annotated ORFs in a publicly available resource (http://genomics.nimr.mrc.ac.uk/online/xt-fl-db.html, assembly Xl4) using methods described previously (Gilchrist et al., 2004); BLASTx alignments between the XGC reference sequences and complete sets of protein sequences from six non-*Xenopus* species; and sequence matching between positionally-defined short sequence tags (96 bp) taken from the start of the XGC reference ORF and similar tags taken from the assembled EST contigs at successive in-frame ATGs from the first. The assembled EST contigs are associated with data that makes use of established full-length scoring methods (Gilchrist et al., 2004), which have recently been refined, and extended with a new *cliff* test to determine the probable start of transcription, incorporating our understanding of the likely behaviour of reverse transcriptase in cDNA library making and its impact on EST sequence data (Gilchrist et al., 2015). The purpose of both the BLASTn alignments and the positional tag matching was two-fold: to identify the EST assembly for the corresponding gene (or alternate homeolog) that each XGC sequence belonged to, and to establish the likely position of the XGC ORF within independently determined open reading frames. See Section 2 for more details.

Applying our tests as described (Section 2), we annotated 7705 (88.8%) of the clones in the *Xenopus* ORFeome (*X. laevis*) as having either full-length ORFs (7396) or ORFs which are technically incomplete but have conserved N-terminus translated sequences (309), starting from an in-frame, conserved ATG. The latter we discuss in detail below, but we expect both of these groups to generate functional proteins. The remainder of the collection is divided into three groups: 493 (5.7%) which gave only negative test results, and which we assume would likely generate functionally impaired proteins; 144 (1.7%) which gave mixed positive and negative indications; and 331 (3.8%) for which no tests were either positive or negative. We refer to these outcomes as *good, conserved*, *bad*, *mixed* and *unknown* respectively (Table 4, Fig. 2b).

**Fig. 3:**
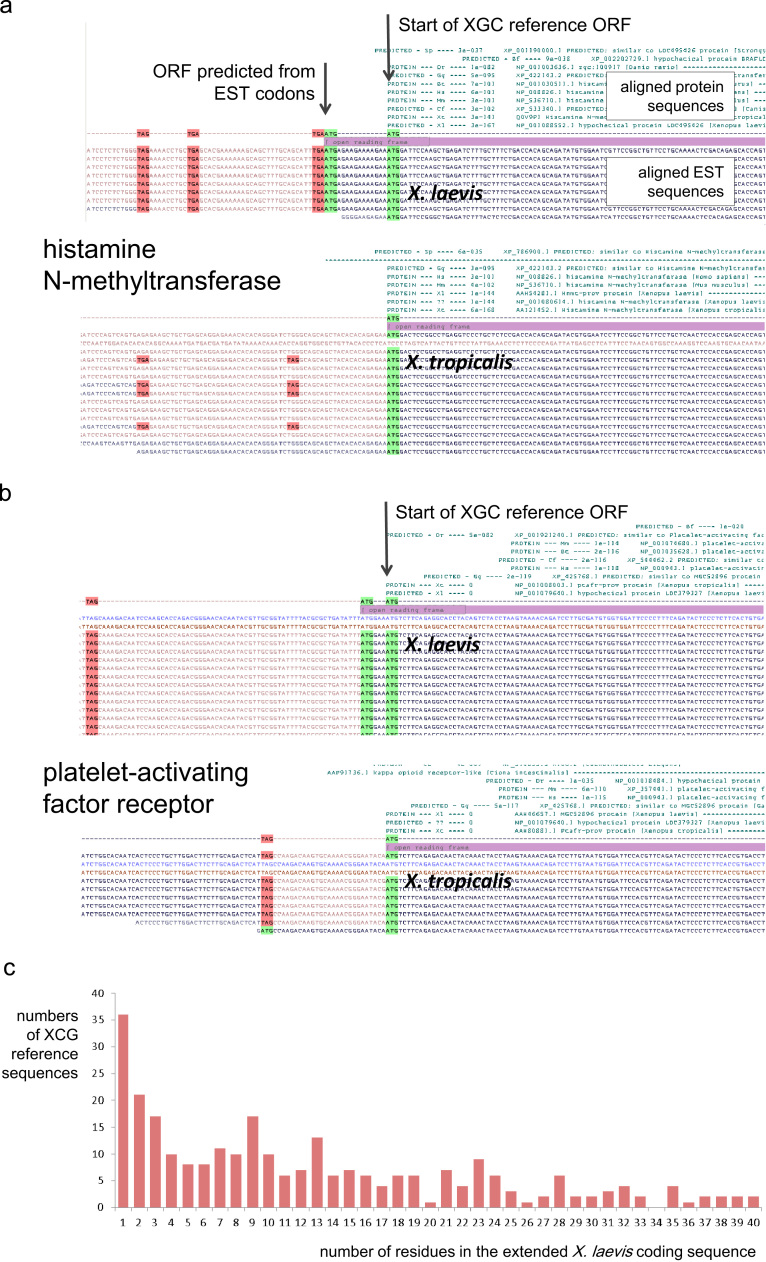
Truncated but conserved ORFS. Examples of genes where the open reading frame in *X. laevis* is slightly longer than in other species, including *X. tropicalis*, suggesting that the shorter ORFeome clone, based on the XGC predicted ORF, is conserved and likely to be functional. Sections of EST clusters showing aligned nucleotide sequences (ESTs) with in-frame stop and ATG codons in red and green respectively. ORF predicted from aligned EST codons is in purple. Start of the XGC reference ORF is indicated by arrow. Protein alignments are shown above in turquoise, with the leftmost ‘P’ being the predicted relative position of the N-terminal end of these proteins. (a) The extremely highly conserved N-terminus of *histamine N-methyl transferase* is extended by 15 bp. (b) The moderately conserved N-terminus of *platelet-activating factor receptor* is extended by 6 bp compared to *Xenopus tropicalis*, and both are longer then the chick, human, mouse and zebrafish proteins. Note the erroneous prediction for the *Xenopus laevis* protein in both cases. (c) Distribution of the numbers of lost N-terminal residues between the shorter, conserved XGC reference ORFs and the longer true ORFs in *Xenopus laevis*.

**Fig. 4:**
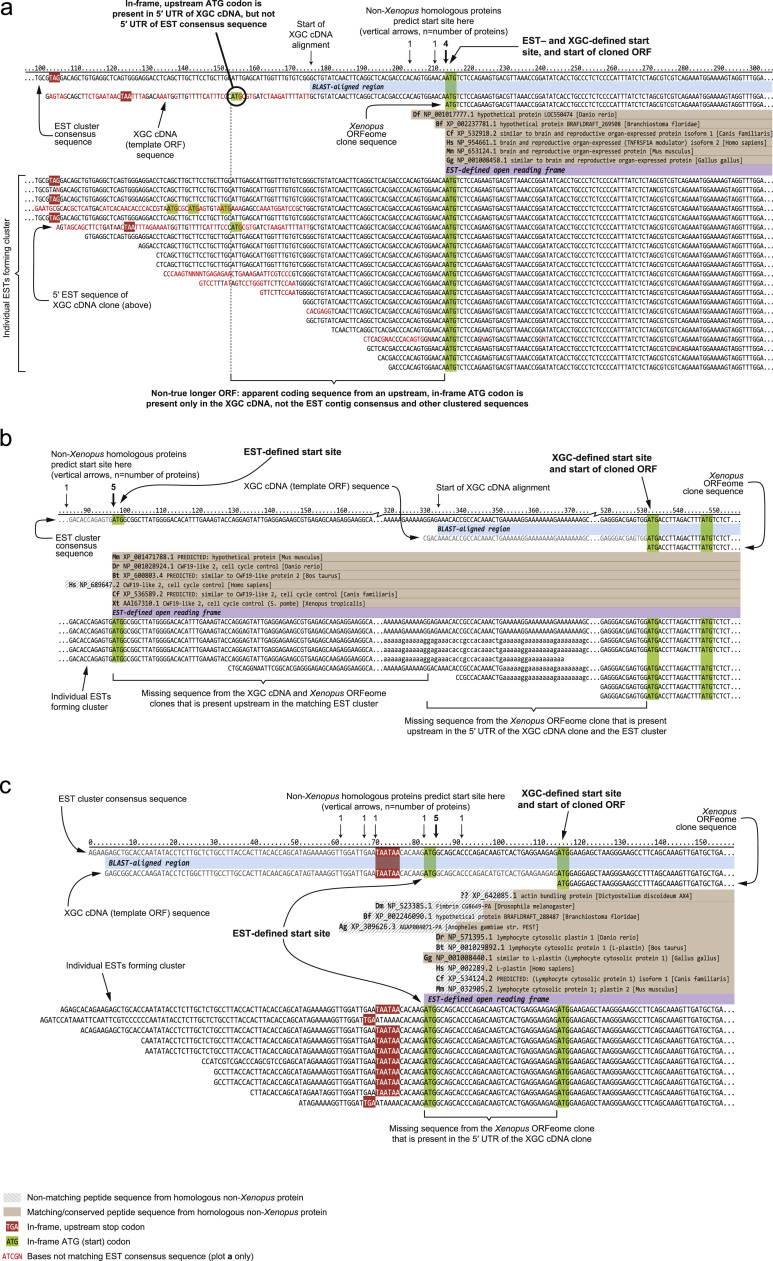
Example alignments of XGC cDNA sequences and matching EST clusters. (a) *Brain and reproductive organ-expressed protein* (*TNFRSF1A*), illustrating divergence between the XGC sequence and the cluster consensus sequence, and adding 60 bp to an upstream ATG. Genomic alignment indicates (data not shown) this to be a non-canonical exon; we note that the XGC prediction was for the canonical start of translation. (b) *CWF19-like 2, cell cycle control* (*cwf19l2*), illustrating 5′ truncation of the entire cDNA sequence (i.e. the ORF and 5′ UTR). The EST cluster and conserved homologous peptide sequences contain additional sequence that is not present in the 5′ UTR of the XGC clone. Some interposing sequence is not shown; the numbered, horizontal scale has been clipped and compressed for clarity. (c) *Lymphocyte cytosolic protein 1* (*L-plastin*), illustrating a 5′ truncated XGC ORF due to erroneous identification of the correct start codon. In this case, the 5′ UTR of the XGC cDNA contains the extra matching coding sequence from the EST cluster, and is supported by matching alignment of conserved homologous peptide sequence from non-*Xenopus* species.

The conserved, but technically not full-length, group is both striking and interesting. These 309 clones share a common feature: the ATG codon defined in the XGC reference sequence as the start of the open reading frame corresponds to the second, or subsequent, in-frame ATG codon in the assembled EST contig data, and this second ATG corresponds more closely to the start of the translated protein in a range of other species including *X. tropicalis*, than the first ATG (Fig. 3a and b). In these cases, therefore, the normal *X. laevis* protein from these loci would contain a small number of additional, non-conserved N-terminal amino acids, and although the effect of this on the function of the protein is unclear, it would seem likely that, in these cases, the slightly shorter protein from the Gateway clone would be fully functional. In many of these cases the XGC reference sequence contains the upstream ATG, but this had not been identified as the start of translation; i.e. it is located in the nominal 5′ untranslated region (UTR). We believe this may stem from the original XGC analysis which allowed full-length prediction on single sequences using protein alignments from other species (Gerhard et al., 2004, Morin et al., 2006). The merit of this approach is that it may avoid mis-prediction in cases where an upstream in-frame ATG is introduced by non-canonical transcription start sites (Fig. 4a) or splicing, or frame-shifts caused by sequencing or cloning errors.

In some of the cases that we studied, it was the upstream ATG in the assembled EST contig that was unreliable, and therefore a fault of the EST-based analysis, not the XGC prediction; this accounted for at least the five most distant upstream ATGs in this group. The distribution of the lengths of the ‘lost’ sequences (Fig. 3c) shows that most of them are short, and in the majority (50.5%) of cases involves a loss of ten or fewer residues.

To confirm the negative analysis we inspected a number of the clones in the *bad* category. These generally fell into two groups: those where the XGC sequence contained an upstream in-frame ATG, which either had better evidence to support the longer ORF (Fig. 4c), or where there was insufficient protein alignment evidence and the longer ORF was logically correct; and those where the XGC sequence appears to be truncated, relative to the EST contig data, and therefore does not contain the upstream, and probably, correct ATG (Fig. 4b). The former (and larger) group will be rescued from the existing clones by designing new 5′ primers where the XGC sequence contains the start of translation, in the correct frame and position, predicted from the EST contig data. In the interest of timely release of v1.0 of the ORFeome, this will be done in the next stage of the project. This will also be done for full ORFs in the *conserved* category described above.

The complementary situation occurs when the XGC reference sequence has an in-frame, but apparently incorrect, ATG upstream of the defined start of the coding sequence, generated by atypical transcription (Fig. 4a) or sequencing and cloning artefacts. In these cases the XGC ORF prediction is likely correct, and we note that these cases support the use of conservation as a criterion for full-length prediction, rather than strict codon-based analysis, when analysing single sequence data.

### 3.5 Recording and updating the full-length-status of clones in the collection

The full-length status of each clone in the *Xenopus* ORFeome is given both in the plate map of the collection (Supplementary Information, Table S1) and in the ORFeome section of Xenbase (www.xenbase.org/reagents/static/orfeome.jsp). The status of individual clones may change in the future as new data resources, such as improvements in the *X. laevis* genome assembly, become available, or as analysis methods are further refined. Data sources will be updated periodically to reflect this: new plate maps will be issued with releases of future versions of the ORFeome, or to coincide with significant improvements in analysis techniques, and data will be updated on Xenbase as frequently as feasible. We suggest that potential users of this resource carefully explore the underlying reference data for their clones of interest, especially where the analysis is ambiguous. In this context it may be important to bear in mind that some *X. laevis* proteins found in public repositories are likely to have been defined from the XGC reference sequence, and we suggest that this data on its own may be insufficient to establish the veracity of the published ORF. Users are welcome to send us evidence for clones they consider to have been wrongly classified, and we will modify our records where appropriate.

### 3.6 Modelling human diseases

To facilitate the use of the *Xenopus* ORFeome resource in experiments aimed at modelling human disease in *Xenopus*, we have identified those genes in the collection whose human orthologs are, either directly or indirectly, implicated in disease; these may also prove useful starting points for functional investigation. To do this we used two sets of human gene–disease association data: the first set was taken from OMIM (http://omim.org) which we count as primary or direct gene–disease associations; the second set was taken from published data (Lage et al., 2008), incorporating OMIM disease genes but extending the disease association to other genes whose proteins are predicted to be in physical complexes (derived from protein interaction data) with OMIM entries, which we count as indirect gene–disease associations. Direct and indirect disease associations are reported in separate columns in the supplementary data file (Supplementary information, Table S2).

To link the human disease genes to our *Xenopus* Gateway clones we used the *Xenopus*–human orthology data available in Xenbase (see Section 2). This also gives us a general estimate of the coverage of human genes within the *Xenopus* ORFeome v1.0, identifying human orthologs for 80% (6940) of the clones, corresponding to approximately 29% of human protein-coding genes.

Combining the direct and indirect disease associations, we have identified 3048 clones in our collection, covering 2724 *Xenopus* orthologs of human genes associated with a total of 2030 different disease disorders (see Supplementary information, Table S2). Of the 2030 human diseases, 1501 (75%) were linked by a direct association to an ORFeome clone, with the remaining 529 having one or more indirect associations. Looking more closely at the coverage of the 1524 complexes defined in the protein interaction data (Lage et al., 2008), we found one or more *Xenopus* ORFeome genes, with a direct or indirect association, in 1349 complexes, covering 974 disease conditions. For 72 of those complexes, all the genes from the complex are present as orthologs in the ORFeome. This high coverage serves to validate the *Xenopus* ORFeome as an invaluable tool for probing the mechanisms underlying many human diseases using the *Xenopus* model system.

### 3.7 Release and distribution of the *Xenopus* ORFeome clones and associated plasmids

The *Xenopus* ORFeome collection is released as of the date of writing. Information on the ORFeome collection including quality assessments of the full-length clones and ordering information is available at Xenbase (www.xenbase.org/reagents/static/orfeome.jsp). To maximise availability to the community we have provided complete sets of plates to a number of distributors, both commercial and not-for-profit. These currently include: Source Bioscience (www.sourcebioscience.com), GE Healthcare–GE Lifesciences/Dharmacon (dharmacon.gelifesciences.com), DNASU Plasmid Repository at the ASU Biodesign Institute (dnasu.org/DNASU/Home.do), Dana-Farber/Harvard Cancer Centre DNA Resource Core (dnaseq.med.harvard.edu), and The European *Xenopus* Resource Centre (EXRC) (www.port.ac.uk/research/exrc). Some of these (e.g. EXRC) may only distribute small numbers of individual clones. In addition Xenbase hosts a search facility under Reagents and Protocols tab for finding ORFeome clones (www.xenbase.org/reagents/orf.do), and users will find links to clones on individual gene pages.

A large number of destination vectors are already available to researchers; we have generated three vectors for the *Xenopus* community based on pCSf107mT, which has a pCS2 backbone and three tandem SP6 stop sequences, circumventing the need to linearise the plasmid when transcribing in vitro (Mii and Taira, 2009). The generation of pCSf107mT-Gateway-3′LAP Tag is given in Section 2; similar methods were used to generate pCSf107mT-Gateway-3′MycTag, pCSf107mT-Gateway-3′HA Tag, and pCSf107mT-Gateway-3′Flag Tag vectors. To increase the accessibility of these four constructs we have submitted them as plasmids to Addgene (www.addgene.org), where they will be available in the normal way (the relevant Addgene IDs are: 67616, 67617, 67618 and 67619). They are currently also available through the EXRC at Portsmouth, UK.

## 4 Conclusions

**Fig. 5:**
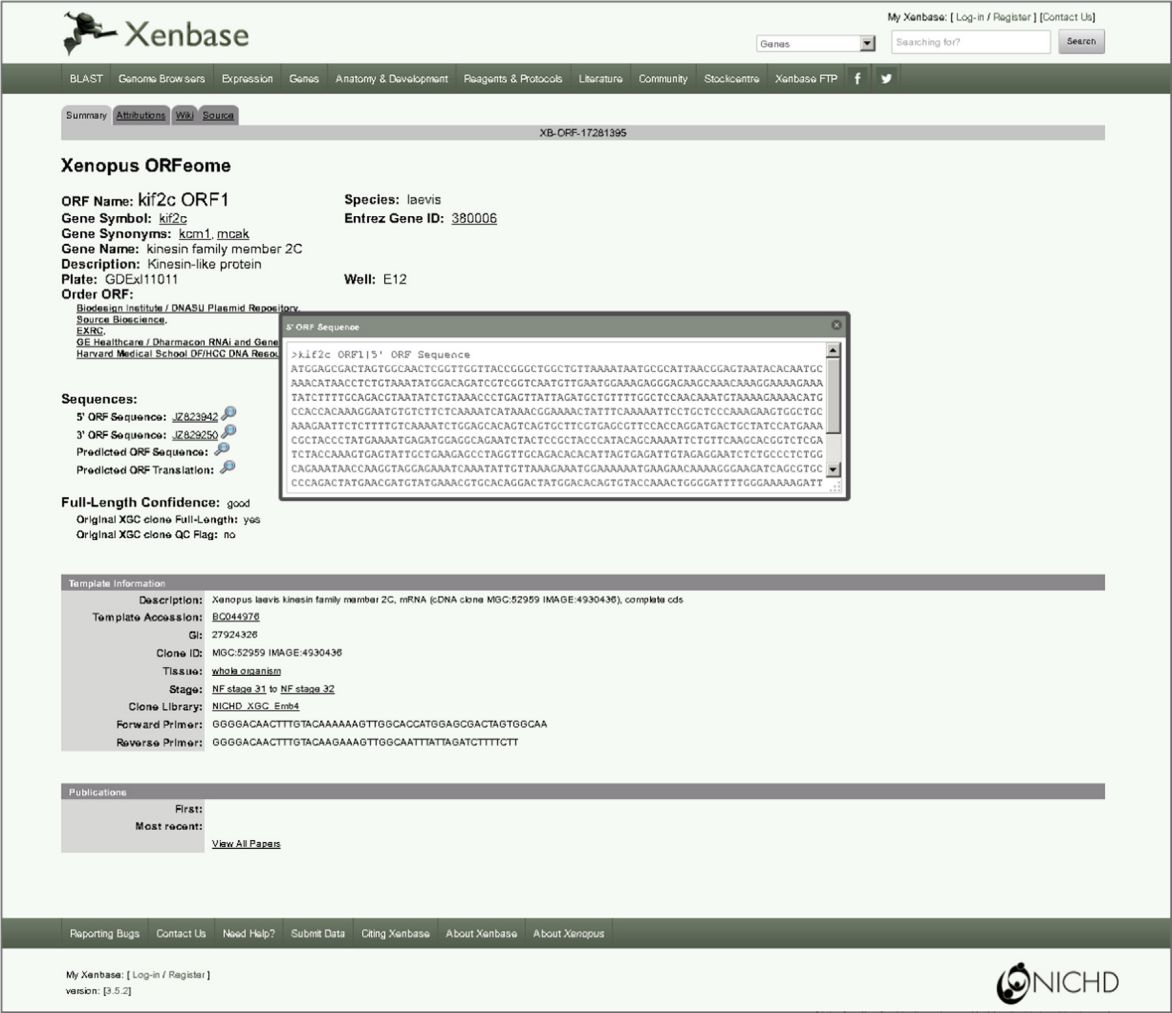
Example screenshot of a web page on Xenbase.org for *Xenopus* ORFeome clone (for Kif2c ORF1), illustrating metadata information such as associated IDs, plate & well location, links to vendors, coding sequence, full-length quality indication, and XGC template cDNA source information.

We have generated v1.0 of the *Xenopus* ORFeome within the Gateway recombination cloning system, using 8673 pre-existing *X. laevis* cDNA clones from the *Xenopus* Gene Collection, and covering an estimated 7871 unique genes. Presented in Gateway entry vectors (pDONR223), they are available to the community to buy as a complete set of 93, 96-well plates, or as individual clones. The *Xenopus* ORFeome resource will be supported on Xenbase.org, the *Xenopus* model organism database; providing links to vendors, and to sources of community developed Gateway destination vectors. Links to ORFeome clones will be found on gene pages, and users can search for clones on the Xenbase ORFeome project page (http://www.xenbase.org/reagents/static/orfeome.jsp), or by BLAST via the gene pages. Each clone has a dedicated page, including NCBI Entrez and RefSeq IDs, the XGC template, PCR primers used to amplify the ORF, and its full-length status (Fig. 5), integrated into Xenbase, alongside other genomic, expression, function and human disease model metadata relevant to each ORF.

The bioinformatic analysis of each clone forms a distinctive component of the *Xenopus* ORFeome: the summary status of each clone is given, the reasoning behind which can be deduced from the individual test scores. This will significantly increase the utility of the collection, and, to the best of our knowledge, is unique amongst ORFeomes. We recommend that users note the full-length status of their clone(s) of interest, and also check the associated reference sequence, especially where our full-length confidence is moderate or low. Version 2.0 of the *Xenopus* ORFeome will use the bioinformatic analysis up front to exclude clearly problematic clones, whilst continuing to include conserved clones, and those where analysis remains unclear.

This version 1.0 of the *Xenopus* ORFeome covers nearly 40% of the *X. laevis* proteome, and currently represents the third largest ORFeome behind the human and *C. elegans* projects. The next release of the *Xenopus* ORFeome will extend gene coverage by including clones from two *X. tropicalis* EST collections (Gilchrist et al., 2004, Klein et al., 2002). The second phase of the project will generate Gateway clones by PCR of missed and computationally defined ORFs from suitable cDNA libraries.

The experimental advantages of *Xenopus* have been used for decades to discover fundamental mechanisms of cell and developmental biology. The recent sequencing of the two *Xenopus* genomes has shown a remarkable similarity with the human genome, including the *Xenopus* orthologs of many human disease genes (Hellsten et al., 2010). The high degree of anatomical similarity, and the ability to easily perform both gain- and loss-of-function studies, makes *Xenopus* ideally suited to studying gene function in models of human disease. Recent examples of the effectiveness of this approach include cancer (Chernet and Levin, 2013), wound healing (Soto et al., 2013), congenital heart disease (Fakhro et al., 2011), and epilepsy (Bell et al., 2011). Coupled with the rapid and cost effective functional genomics possible in this system, the *Xenopus* ORFeome collection will greatly facilitate a mechanistic analysis of the proteome and accelerate our understanding of human disease.

## Figures and Tables

**Table 1:** Comparison of other Gateway-cloned ORFeome projects.

**ORFeome**	**Source of ORFs**	**Clones**	**Non-redundant genes**	**Estimated proteome coverage**
**hORFeome v8.1 [*****H. sapien*****]**	Existing ORFs from MGC cDNA library extracted via PCR	16,172	13,833	73% Yang et al.(2011)
***M. musculus***^a^	DNA synthesis of coding sequences supported by known transcripts and protein orthologs	3414^a^	1349^a^	7.0%^a^Temple et al. (2009)
***C. elegans*****v1.1**	Known or predicted ORFs from GenBank, the Transcriptome project, or WormBase amplified by PCR from a cDNA library	11,984 (10,623 usable)	11,984 (10,623)	62% (55%) Reboul et al. (2003)
***S. cerevisiae***	Known ORFs amplified by PCR from genomic DNA	5854	5854	93% Gelperin et al. (2005)
***B. melitensis*****v1.1**	Genome-annotated ORFs (with some manual correction) were used in PCR amplification from genomic DNA	3091	3091	97% Dricot et al. (2004)
***C. pneumoniae***	Genome-annotated ORFs were used in PCR amplification from genomic DNA	1037	1037	99% Maier et al. (2012)
***S. aureus***	Genome-annotated ORFs were used in PCR amplification from genomic DNA	2562	2562	95% Brandner et al. (2008)
***E. coli***	3734 existing ORFs transferred directly from ASKA plasmid library, ∼250 amplified via PCR from genomic DNA	3974	3974	94% Rajagopala et al. (2010)
***D. melanogaster*****UAS-ORFeome**	ORFs cloned from *Drosophila* gene collection or Berkeley *Drosophila* Genome Project cDNA libraries via PCR	1149	1149	8.5% Stapleton et al. (2002)
***Xenopus*****ORFeome v1.0 [*****X. laevis*****] (this work)**	Existing ORFs from XGC cDNA library extracted via PCR	8673	7871 (8402 homeologs)	39% (28%) Gilchrist (2012)

a Only a minority of full-length mouse cDNA clones were created as Gateway entry clones; the entire Mammalian Gene Collection contains full-length clones for 89% (17,704) of mouse genes**.**

**Table 2:** Tests of full-length validity were applied to each of the XGC ORFs. If a clone fits the criteria, the score is +1 for that test; otherwise the score is 0. The suffix *.p* denotes a positive test, i.e. fitting the criteria for such a test is a positive indicator for the ORF being truly full-length; .*n* denotes a negative test – matching these tests indicates the ORF may not be full-length. See Fig. 2a for definitions of C,P,A.

**Test**		**Description**
**1.*****p***	ATG word correct	The ATG word (12 bases at the beginning of the ORF) on the XGC cDNA matches that from the best-hit EST cluster.
**2.*****p***	$\bar{P}\approx A$	The average predicted start codon position on the cDNA from non-*Xenopus* protein alignments ($\bar{P}$) is within 12 bp of that defined by the XGC ORF (A).
**3.*****p***	∑(P≡A)≥2	If at least two separate, individual protein alignments from non-*Xenopus* species give P=A*exactly*, then this is good evidence of veracity, due to low probability of this occurring by chance.
**4.*****p***	Positive tag match	The 96 bp tag from the start of the XGC ORF matches a similar tag from the Xl4 EST assembly marked ORF, CONS, CLI, or ATG1, implying that the XGC start codon matches the start of the open reading frame or a conserved N-terminus translation.
**5.*****n***	ATG codon upstream in 5′ UTR matches EST	The EST and cDNA ATG words do not match. When the sequences are aligned, the relative position of the EST start codon is upstream of the cDNA start codon. This means that the cDNA start codon matches another ATG codon downstream of that defined as the start codon on the EST cluster sequence, i.e. the cDNA ORF is truncated with respect to the EST ORF at the 5′ end. In this case, an upstream ATG codon in the 5′ UTR of the cDNA matches that defined as the start codon on the EST, i.e. this is possible evidence that the XGC ORF has been incorrectly defined using a downstream ATG codon.
**6.*****n***	$\bar{C}\sim 0\bar{P}<<0$	Protein conservation includes the 5′ UTR of the XGC cDNA sequence ($\bar{C}<15$ or A2 where the 5′ UTR is very short), but the typical length of the conserved protein is longer, resulting in a negative value for $\bar{P}$ (we used a cut-off of $P_{\max}<-15$ or $\bar{P}+\sigma_{P}<-15$) indicating that the full cDNA sequence (ORF+5′ UTR) is truncated at the 5′ end.
**7.*****n***	Negative tag match	The 96 bp tag from the start of the XGC ORF matches a similar tag from the Xl4 EST assembly marked ATG2, ATG3, or greater (up to ATG9) implying that the XGC start codon matches an in-frame ATG partway into the open reading frame, and is not likely to represent a conserved N-terminus translation.

**Table 3:** The XGC clones were classified according to our predictions as to the veracity of their ORFs based on the sum of scores from the tests defined in Table 2.

**Full-length status**	**Definition from test scores**	**Description**
**Good**	$\sum_{i=1}^{4}i.p>0,\;\sum_{i=5}^{7}i.n=0$	At least one of the positive tests was passed, and none of the negative tests were failed; the ORF is highly likely to be full-length.
**Conserved**	4.p=1,tag=CONS	Test **4.*****p*** applies (scores +1), and the tag type (‘CONS’) indicates the presence of a conserved ATG (N-terminus); this will override other negative tests. Even though the ORF is technically truncated the protein is likely to be functional.
**Mixed**	$\sum_{i=1}^{4}i.p>0,\;\sum_{i=5}^{7}i.n>0$	Passed at least one positive test, but also failed at least one negative test; the ORF is probably still full-length, but the data are inconclusive.
**Bad**	$\sum_{i=1}^{4}i.p=0,\;\sum_{i=5}^{7}i.n>0$	The clone passed none of the positive tests and failed at least one of the negative tests; the ORF is likely to be wrong based on comparison with EST and conserved protein data.
**Unknown**	$\sum_{i=1}^{4}i.p=0,\;\sum_{i=5}^{7}i.n=0$	No tests were passed or failed; the data are insufficient to determine whether the ORF is full-length or not.

**Table 4:** Classification of full-length status of the ORFs in the *Xenopus* ORFeome.

**Full-length classification**	**Clones in*****Xenopus*****ORFeome v1.0**
**Good**	7396 (85.3%)
**Conserved**	309 (3.6%)
**Bad**	493 (5.7%)
**Mixed**	144 (1.7%)
**Unknown**	331 (3.8%)
